# Strength and Fracture Toughness of TIG- and Laser-Welded Joints of Low Carbon Ferritic Steels

**DOI:** 10.3390/ma17163956

**Published:** 2024-08-09

**Authors:** Tadeusz Pała, Wiktor Wciślik

**Affiliations:** 1 Faculty of Mechatronics and Mechanical Engineering, Kielce University of Technology, Al. 1000-lecia PP 7, 25-314 Kielce, Poland; 2 Faculty of Civil Engineering and Architecture, Kielce University of Technology, Al. 1000-lecia PP 7, 25-314 Kielce, Poland; wwcislik@tu.kielce.pl

**Keywords:** 13CrMo4-5, 16Mo3, welding, strength properties, fracture toughness, FEM modelling

## Abstract

This paper presents the results of experimental testing of joints welded using conventional TIG and laser methods. The welded components were sheets of the low-carbon steels 13CrMo4-5 and 16Mo3. Welded joints were made using different levels of linear welding energy. In the case of laser welding, a bifocal beam with longitudinal positioning of the focal lengths in relation to the welding direction was used. Experimental tests on welded joints included a bending test and determination of hardness distribution, mechanical properties, and fracture toughness, as well as microstructural research in the material of the various joint zones. Based on the determined strength characteristics, the true stress–strain relationships were defined, and a numerical model of the laser joints was developed in Abaqus 6.12-3. The modelled joint was subjected to loading to determine the most stressed areas of the joints. The numerical results were compared with those obtained using GOM’s Aramis 3D 5M digital image correlation system. The system used made it possible to record displacements on the surface of the analysed joints in real time. Good agreement was obtained between the strain fields calculated numerically and those recorded using the Aramis 3D 5M video system. The numerical calculations provided information on the strains and stresses occurring inside the analysed joint during loading. It was found that the welded joints were characterised by increased hardness and high strength properties in relation to the base material. The bending test of the laser-welded joints gave a positive result—no cracks were observed on the face or root of the weld. The fracture toughness of the joint zones is slightly lower in relation to that of the base material, but no brittle fracture was observed.

## 1. Introduction

Welding steel elements plays a key role in the design of various types of structures. Welding is a complex process during which transformations in the material take place. They depend on the choice of welding method and parameters, as well as the preparation of the elements [1,2,3]. The quality of the welds often determines the durability and operational reliability of certain objects. In the energy industry, such facilities include power boilers [4,5]. The type of steel grade used for a particular power plant depends on the operating conditions, such as temperature and pressure, for example [6,7,8,9]. One of the basic requirements for materials used in the manufacture of power boilers is their weldability and high creep resistance [10]. These requirements are met by the steels 13CrMo3-4 and 16Mo3. These steels are traditionally welded using a combination of TIG and MMA and SMAW welding methods [11,12,13]. Recently, there has been increasing interest in laser welding, which has a number of advantages, including high productivity and no need for additional material. Laser welds are characterised by their narrow weld and heat-affected zone [14,15].

However, during laser welding, material transformations take place that lead to an increase in hardness in the weld material (WM) and heat-affected zone (HAZ). Additionally, an increase in strength characteristics, levels of factor *M = σ*_yw_*/σ*_yb_ (where σ_yw_ and σ_yb_ are the yield strength of the weld material and the base material, respectively) [16], and residual stress in the joint are observed. When welding modern steels with bainite and tempered martensite microstructures in the vicinity of the WM and HAZ, reduced strength characteristics and increased material brittleness were observed [17,18]. Appropriate selection of welding parameters, especially the reduction of linear welding energy (LWE) levels, makes it possible to obtain joints characterised by high strength.

Laser welding in the WM and HAZ of 13CrMo3-4 and 16Mo3 steels also produces bainite and tempered martensite microstructures, which can have an unfavourable effect on ductility and fracture toughness. Therefore, this article presents the results of a comprehensive study, the aim of which was to accurately determine the characteristics of strength, ductility, and fracture toughness in the WM and HAZ of laser joints, as well as an assessment of the stress state in the various joint zones during loading carried out by means of numerical simulation. For comparison purposes, the article presents the results obtained on the material of joints made using the conventional TIG method.

## 2. Base Material—Specification of Welding Technology

13CrMo4-5 and 16Mo3 steels are widely used for the construction of power equipment such as boilers, tanks, and gas and steam turbine components [19,20]. They are characterised by good weldability and high corrosion and creep resistance and are suitable for high-temperature service (up to 550 °C) [21]. These steels are distinguished by their high plasticity and ductility, both at low and high operating temperatures. The mechanical properties and chemical compositions of the welded steels are presented in Table 1 and Table 2, respectively.

**Figure 1:**
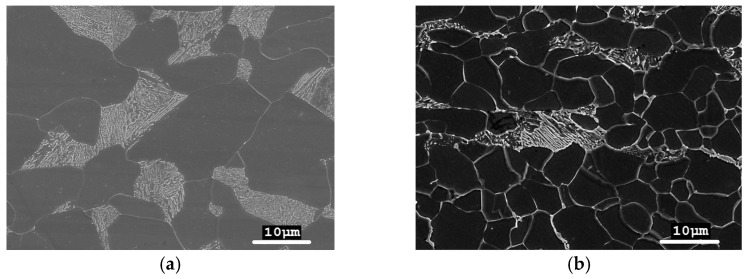
Ferritic-pearlitic microstructure of steel: (**a**) 13CrMo4-5; (**b**) 16Mo3.

Steel grades 13CrMo4-5 and 16Mo3 have a ferritic-pearlitic microstructure with a ferrite grain size distribution of 5–25 µm (Figure 1). In the microstructure of the 16Mo3 steel, a distinct banding of pearlite areas was observed. A lamellar structure was visible in the pearlite colonies.

**Figure 2:**
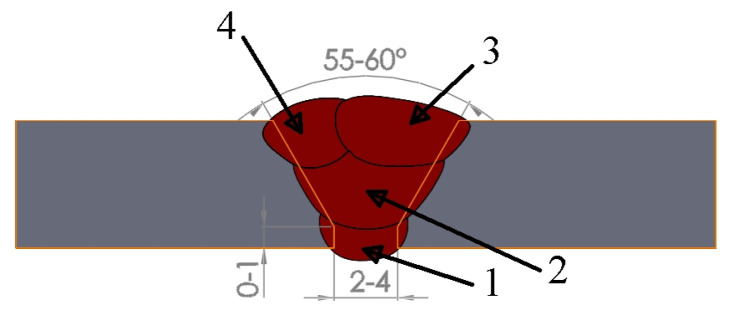
Scheme of the welding groove and preparation of a TIG weld. Symbols 1–4 indicate the bead number.

The base materials in the tested joints were 13CrMo4-5 and 16Mo3 steel plates with thicknesses of 6.7 mm and 6.0 mm. The basic parameters for conventional TIG and laser welding are presented in Table 3 and Table 4, respectively. In the case of conventional TIG welding, the edges of the sheets to be joined were suitably prepared by milling (Figure 2). The differences in laser joint technology are due to the welding speed and the shielding gas used (Table 4). It should be noted that the TIG-welded joints were made using much higher LWE than the laser-welded joints for both steel grades. Figure 2 shows how the individual beads were arranged, in the order in which they were made. Before welding 13CrMo4-5 steel using the TIG method, the sheets were preheated to a temperature of 225 °C. A DCMS-IG welding rod for 13CrMo4-5 steel and DMO-IG for 16Mo3 steel were used as additional materials. Argon was used as a shielding gas.

In the case of laser welding, the edges of the joined sheets were milled without bevelling. The joints were made with one pass of the laser beam without the use of additional material or preheating. The welding process was carried out using a CO_2_ laser equipped with a bifocal head. A laser beam with a wavelength of 10.6 µm and a pulse frequency of 50 kHz was emitted. Focusing was applied along the welding direction. The centres of the laser beams were set at a distance of 0.7 mm from each other. The lengths of both focal lengths were 200 mm, and the spot sizes were 200 µm. The beams were characterized by TEM_01_.

## 3. Results

### 3.1. Hardness Distributions and Bending Test

**Figure 3:**
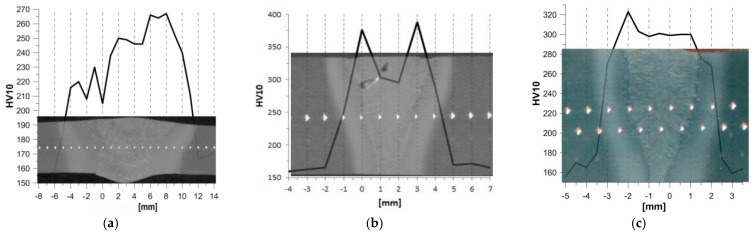
Hardness distributions against the weld background for joints made of 13CrMo4-5 steel: (**a**) TIG (1 T); (**b**) laser 1 L; (**c**) laser 2 L.

Hardness measurements in the analysed welded joints were conducted in accordance with the standard described in [22]. A Wolpert Wilson Instruments 310 HVS hardness tester with a Vickers indenter was used to carry out the measurement. The hardness distributions in the TIG-welded (1 T) and laser-welded 1 L and 2 L joints of 13CrMo4-5 steel were determined along a measurement line located in the central part of the joint along the thickness (Figure 3).

The weld in the TIG-welded joint (1 T) shown in Figure 3a is characterised by its considerable width—on the face side it is 12 mm, then decreases in the middle thickness of the joint to a value of about 6 mm, before reaching a value of 7 mm on the root side. The width of the HAZ is equal to about 4 mm. From the hardness distribution diagram for a conventional TIG (1 T) joint, it can be seen that the weld material is characterised by a hardness of 205–265 HV10, then the hardness gradually decreases with increasing distance from the weld to a value of 185–265 HV10 to finally reach a hardness corresponding to the base material of 155–170 HV10.

Laser joints are characterised by a narrow weld and HAZ. In the 1 L laser joint (Figure 3b) made with LWE = 540 kJ/m, the width of the weld equals ~5.0 mm at the face side then narrows to 3.0 mm at the centre of the joint, reaching a width of about 1.0 mm at the weld root. The width of the HAZ is ~1.1 mm, with a slight reduction at the weld face.

The 2 L laser joint (Figure 3c) made with LWE = 463 kJ/m has a weld width on the face side of approximately 3.5 mm, where it narrows slightly in the middle of the joint thickness to 3.0 mm. After reaching the middle of the joint thickness, the width of the weld material narrows towards the root to 0.7 mm.

In the laser joints, increased hardness was observed in the WM and HAZ. In the 1 L laser joint, a hardness of 300 HV10 is observed in the central zone of the WM, then the hardness increases to about 380 HV10 in the FL (fusion line) zone, then a decrease in hardness to a level of about 250 HV10 in the HAZ is observed and the BM hardness level of 155 HV10 is reached. The 2 L laser joint made with the lower LWE is characterised by a less differentiated level of hardness values found in the WM (300 HV10) and the material found near the FL (320 HV10).

**Figure 4:**
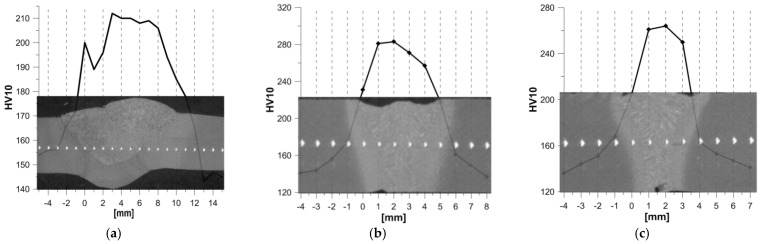
Hardness distributions against the weld background for joints made of 16Mo3 steel: (**a**) TIG (2 T); (**b**) laser 3 L; (**c**) laser 4 L.

Figure 4 shows the hardness distributions for the TIG joint (2 T) and the 3 L and 4 L laser joints, where the BM was 16Mo3 steel.

A joint welded using the TIG (2 T) method is characterised by a wide weld, which is ~13 mm on the face side, then narrows in the middle of the thickness of the joint to 6 mm, and finally reaches a width of 7 mm at the root. The average width of the HAZ in the joint is approximately 3 mm. In this joint the WM has a hardness of ~210 HV10, then through the HAZ the hardness gradually decreases to ~150 HV10 in the BM.

For a 3 L laser joint made at LWE = 540 kJ/m, the width of the weld on the face side is equal to 5.5 mm, narrowing to ~3.0 mm in the centre, reaching a value of ~2.5 mm at the root (Figure 4b). The width of the HAZ is similar, ~1.2 mm, along the height of the weld. The highest hardness occurs in the WM (~280 HV10), and in the centre of the HAZ the hardness decreases to ~220 HV10.

In the 4 L laser joint, for LWE = 405 kJ/m at the face side the HAZ has a width of ~4.0 mm, and in the middle of the joint height it narrows to 2.0 mm, reaching a width of ~1.5 mm at the root (Figure 4c). The width of the HAZ is ~1.0 mm throughout the height, with a slightly smaller width at the face. The hardness of the WM reached ~265 HV10, with approximately 190 HV10 at the middle of the HAZ.

**Figure 5:**
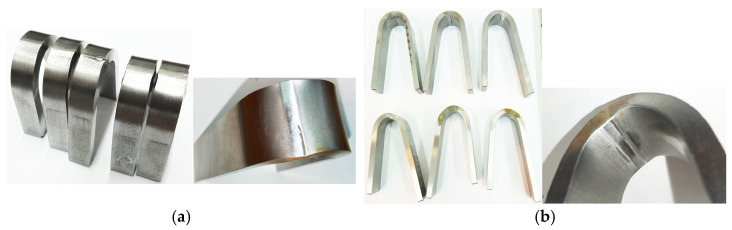
Example photos of specimens after the bending test: (**a**) TIG joint (2 T); (**b**) laser joints 3 L, 4 L in 16Mo3 steel.

The bending test was made according to the recommendations of standard [23] on a Zwick testing machine (100 kN) using a bending mandrel with tension at the root and face of the weld. No cracks were observed in the tested specimens, indicating that the tested joints had adequate ductility and no welding defects (Figure 5). In the pictures of the specimens shown, the darker areas only show undermining of the base material on the sides of the weld. Figure 5 also shows detailed photos of the specimens where undermelting occurs.

### 3.2. Microstructure of the Material in Welded Joint Zones

The hardness changes occurring in the joints due to the microstructural changes of the material are caused by the effect of temperature during the welding process. Microstructural research in different joint zones were carried out for selected welded joints using a JSM-7100F scanning electron microscope. Sections of welded joints were ground, polished, and etched with a 4% solution of HNO_3_ in C_2_H_5_OH (Nital).

**Figure 6:**
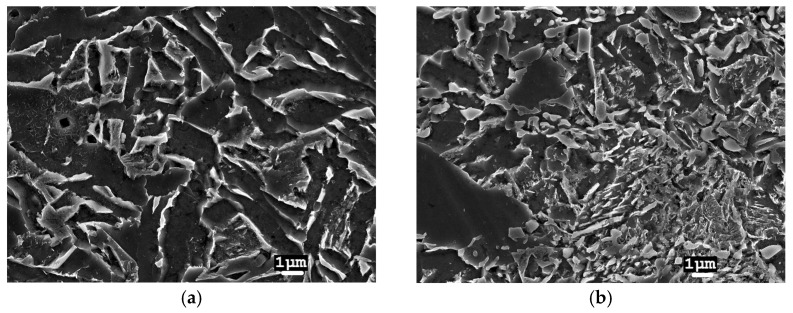
Microstructure of a TIG joint (13CrMo4-5 steel): (**a**) in the WM centre; (**b**) in the HAZ.

In TIG-welded joints, the beads were applied one by one, so the level of total welding energy was high, the temperature effect was relatively prolonged, and cooling was slow. This resulted in the formation of a fine-grained ferrite microstructure in the WM (Figure 6a). In the HAZ material, there was mainly spheroidisation of the pearlite and additional separation of particles from the matrix and their coagulation, resulting in a mixture of bainitic-ferritic type microstructures (Figure 6b).

**Figure 7:**
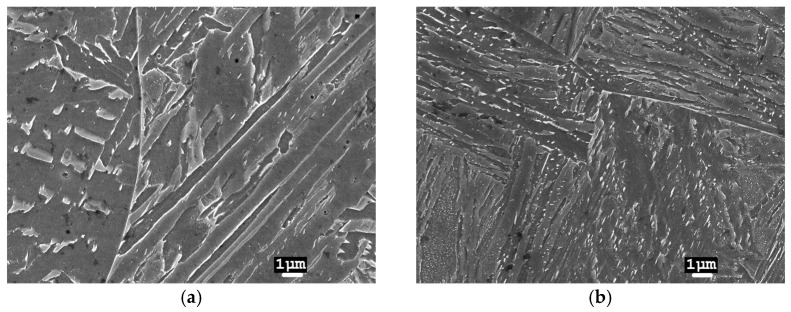
WM microstructure of a 2 L laser joint (13CrMo4-5 steel): (**a**) in the centre of the weld; (**b**) near the FL.

**Figure 8:**
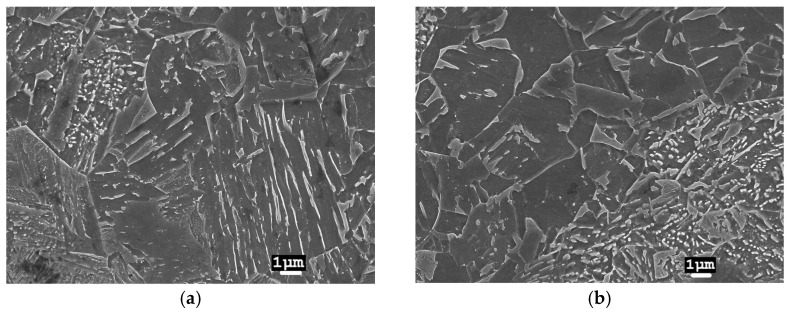
Microstructure of a 2 L laser joint in the HAZ (13CrMo4-5 steel). Distance from weld axis: (**a**) ~2 mm; (**b**) ~3 mm.

In laser-welded joints, the heating and cooling intensities are much higher at lower LWE levels. As a result of the large and rapid changes in temperature effects in WM, a martensitic-type microstructure is formed (Figure 7). In the HAZ closer to the melt, a mixture of martensitic and bainitic type microstructures was observed (Figure 8a), while at the BM, a mixture of bainite and ferrite microstructures was found (Figure 8b). During laser welding, the width of the HAZ is small, and the change in microstructure type is rapid.

### 3.3. Strength and Fracture Toughness of the Material in Different Zones of Welded Joints

#### 3.3.1. Strength Properties

**Figure 9:**
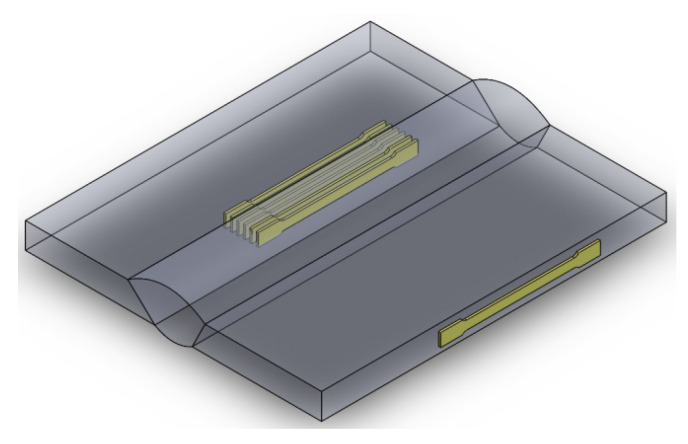
Scheme of cutting specimens for uniaxial tensile testing from a joint.

The strength and ductility of the material in the various joint zones were determined by uniaxial tensile testing on flat specimens with cross-sectional dimensions of 0.5 × 2.0 mm. Specimens were cut from the joints parallel to the welding direction. The specimens for testing are shown in Figure 9. Specimens were taken using electrical discharge cutting (EDM) and a high-pressure water jet. The advantages of the technology used are the lack of thermal effects on the metal and the narrow cutting gap of ~1.0 mm, which allows more specimens to be taken from the joint.

Before carrying out the strength tests, hardness was measured on each specimen. Uniaxial tensile tests were carried out in accordance with the standard described in [24] on the Zwick-100 testing machine with an electrodynamic drive, which is equipped with an automated control and data recording system. Nominal (engineering) and true tensile curves were determined from the tests. Knowledge of the strength characteristics in the individual zones of the analysed joints is essential, as these are used in experimental methods when determining fracture toughness characteristics and in numerical calculations as a material constitutive relationship—the relationship of true stresses and strains.

**Figure 10:**
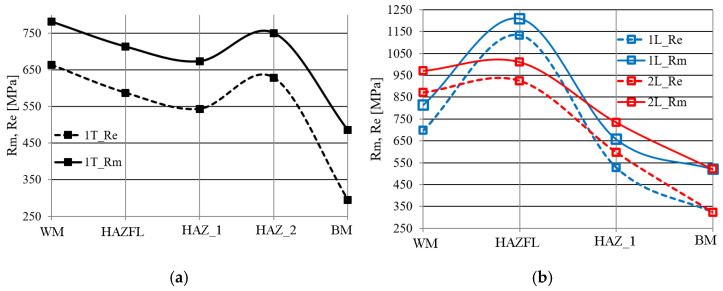
Strength properties of (**a**) TIG and (**b**) laser joint zones (13CrMo4-5 steel).

Strength properties in TIG (1 T)- and laser (1 L, 2 L)-made joints of 13CrMo4-5 steel were determined in the WM, in the vicinity of the FL, in one or two areas of the heat-affected zone (HAZ_1, HAZ_2), and in the BM (Figure 10). In the TIG (1 T)-welded joint, compared to BM, higher strength levels were recorded in all zones. The highest level of strength values is found in the WM, followed by a slight decrease in the FL and HAZ_1 materials. A local maximum is reached in the HAZ_2 material. The lowest levels of strength characteristics were observed in the BM (Figure 10a).

As in the TIG joint, the level of strength properties in laser joints is higher than in the BM (Figure 10b). Maximum strength levels in laser joints were observed near the FL.

The 1 L laser joint (LWE = 540 kJ/m) is characterised by a more dynamic change in strength characteristics compared to the 2 L laser joint (LWE = 463 kJ/m), with a similar change trend. The WM of the joints analysed has more than one and a half times the strength characteristics of the BM and reaches maximum values near the FL, after which a reduction in strength characteristics to the BM strength level was observed in the HAZ material.

Strength properties of similar quality occurred in individual zones of welded joints in which the base material was 16Mo3 steel.

**Figure 11:**
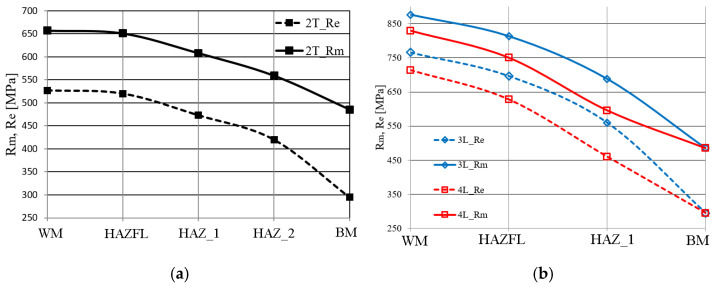
Strength properties of (**a**) TIG and (**b**) laser joint zones (16Mo3 steel).

**Figure 12:**
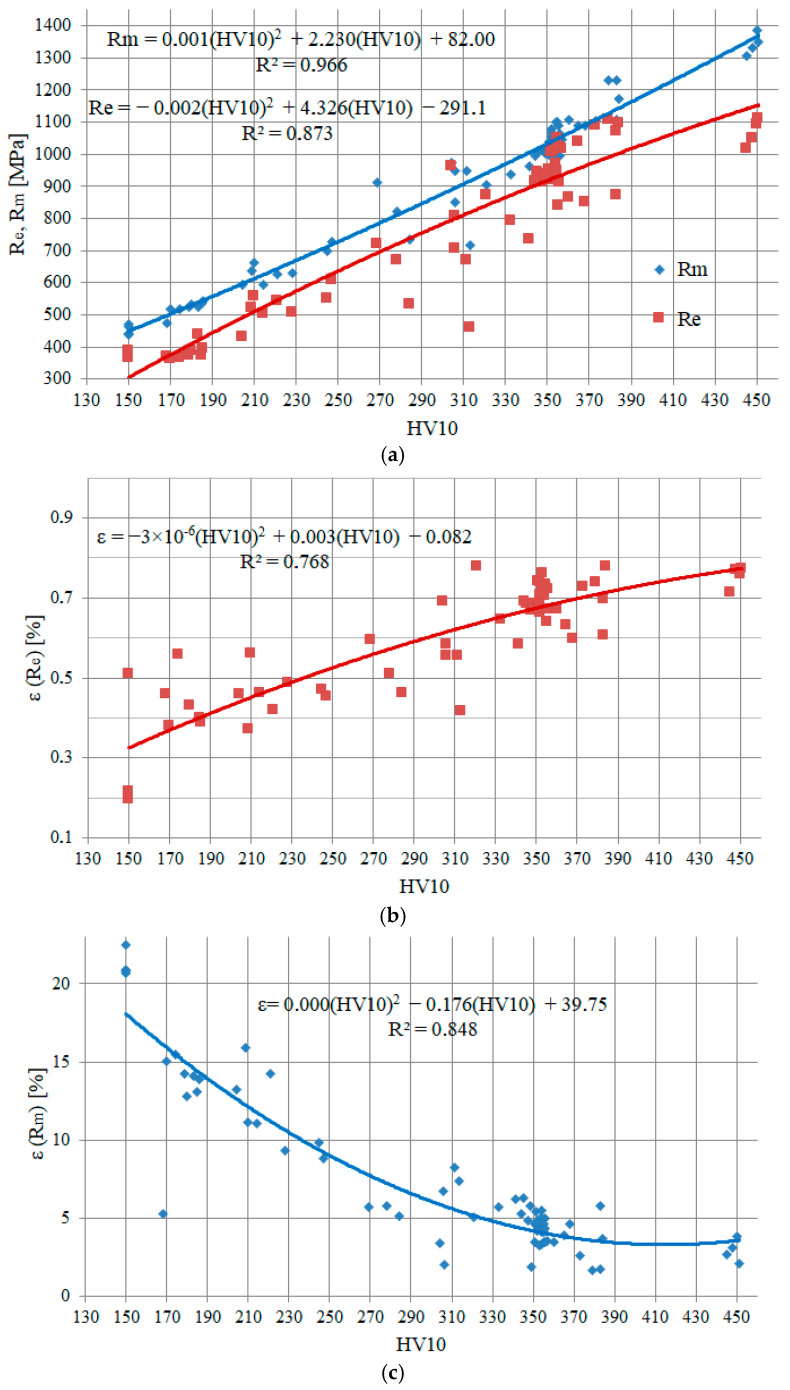
Plots of strength characteristics as a function of HV10 hardness: (**a**) *R*_e_ and *R*_m_; (**b**) strain when *R*_e_ is reached; (**c**) strain when *R*_m_ is reached.

The strength properties in the different zones of the joint made by TIG welding (2 T) and laser welding (3 L, 4 L) are shown in Figure 11. Strength properties were calculated from the determined hardness distribution using the correlation formulae in Figure 12.

Analysing the results obtained, it can be seen that the WM in the analysed joints is characterised by very high strength properties. As the distance from the weld axis increases, a gradual decrease in the strength properties to the strength level of the BM is observed.

Slightly higher strength properties in each zone were observed in the 3 L joint made with LWE = 540 kJ/m with respect to the 4 L laser joint made with LWE = 405 kJ/m (Figure 11b). The TIG joint (2 T) had the lowest strength level in relation to the individual zones (Figure 11a).

The strength characteristics in the welded joint material change smoothly, namely in the HAZ, so it is difficult to take specimens to determine them in all areas. For this reason, in order to determine the values of the strength characteristics in any zones of the welded joints, relationships between hardness and material properties determined during the tensile test were prepared (Figure 12). These relationships allow the values of the relevant characteristics to be calculated from hardness measurements. In the following research, the material dependencies of the true stress–strain used in the numerical calculation of stresses in welded joints were defined based on these formulae.

#### 3.3.2. Fracture Toughness

**Figure 13:**
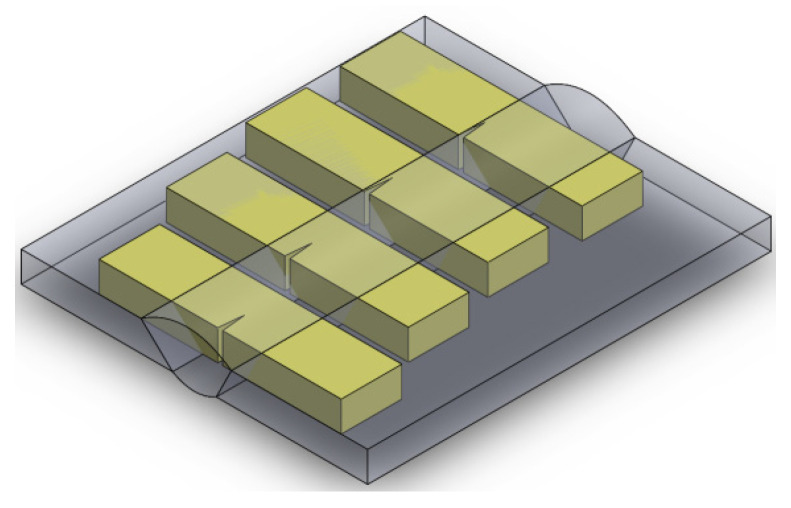
Scheme of specimens and notching of stress concentrators in the different zones of a welded joint.

The fracture toughness characteristics of the material from different welded joint zones were determined using an MTS-250 (250 kN) testing machine equipped with an automated control and data recording system. Fracture toughness tests were carried out on prismatic specimens of the SENB (Single Edge Notch in Bending) type with dimensions *B* = 6 mm, *W* = 12 mm, *S* = 4 *W* with a one-sided crack depth *a*/*W* ≈ 0.5 loaded according to a three-point bending scheme. Fatigue cracks were derived in the WM, in the vicinity of the FL, in the heat-affected zone immediately adjacent to the weld (HAZ_1), in the heat-affected zone at the end of the normalisation zone (HAZ_2), and in the BM, respectively. A schematic of the specimens and notching of the stress concentrators in the welded joint is shown in Figure 13.

The fracture toughness was determined as a critical *J*-*J*_IC_ integral value according to the recommendations of ASTM E1820-15 [25] and then converted to stress intensity factor units according to the formula:

(1) $$K_{JC}=\sqrt{\frac{J_{IC}E}{\left(1-\nu^{2}\right)}}$$

**Figure 14:**
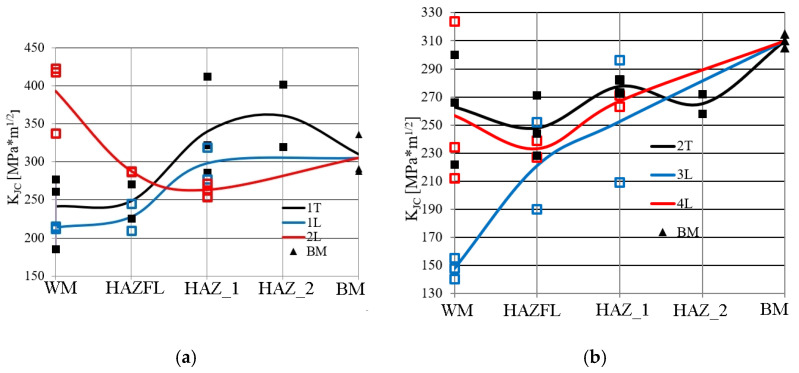
Critical value of fracture toughness in joint zones: (**a**) 1 T, 1 L, 2 L for 13CrMo4-5 steel; (**b**) 2 T, 3 L, 4 L for 16Mo3 steel.

In the SENB specimens taken from the TIG (1 T)-welded joint, there was a ductile crack growth mechanism during loading. The fracture toughness results obtained are shown in Figure 14a. In the case of fracture toughness, the weld material and the material in the vicinity of the fusion line have lower values in relation to the base material, but the level of critical fracture toughness values is well above the value *K*_JC_ = 100 MPa·m^1/2^, which characterises the transition from a brittle to a ductile mechanism [26].

Figure 14a also shows the results of fracture toughness tests in different zones of laser-welded joints. In the 1 L laser joint (LWE = 540 kJ/m), the lowest level of critical value of fracture toughness was recorded in the WM and in the vicinity of the FL. In the HAZ material, the critical value of fracture toughness increases and is slightly lower than that of the BM.

In the 2 L laser joint (LWE = 463 kJ/m), the WM has a high critical value of fracture toughness, which is higher than that of the BM. The lowest critical value of fracture toughness was recorded for the HAZ. However, it should be noted that, in all zones in the 1 L and 2 L laser joints, the critical value of fracture toughness reached high levels (*K*_JC_ > 200 > 100 MPa⋅m^1/2^), indicating the occurrence of ductile fracture.

The results of the critical value of fracture toughness tests in the TIG (2 T) joint and the 3 L and 4 L laser joints of 16Mo3 steel are shown in Figure 14b. The lowest average level of critical value of fracture toughness for the TIG (2 T) joint is found in the material near the FL. Slightly higher average critical value of fracture toughness was recorded in the WM, with the WM zone having the largest scatter in the results obtained on the tested specimens. In the heat-affected zone (HAZ_1) located in the immediate vicinity at the FL, the critical value of fracture toughness increased, followed by a slight decrease in the heat-affected zone (HAZ_2) and finally an increase in critical value of fracture toughness to a level corresponding to the BM. In the zones of a joint made by conventional TIG welding, the level of fracture toughness guarantees the development of a crack according to the ductile mechanism, even though it is lower than the BM.

In laser-welded joints 3 L (LWE = 540 kJ/m) and 4 L (LWE = 405 kJ/m), the lowest critical value of fracture toughness was recorded in WM. In the case of the 3 L joint, the critical value of fracture toughness in WM decreased to *K*_JC_ = 140 MPa⋅m^1/2^ (Figure 14b). As the distance from the weld axis increases, the critical value of fracture toughness level of the individual zones increases, reaching the fracture toughness level for the BM. In the 4 L laser joint made with less LWE, higher critical values of fracture toughness in the WM and HAZ (*K*_JC_ > 200 MPa⋅m^1/2^) were obtained than in 3 L.

### 3.4. Strain and Stress Analysis of Welded Joints

**Figure 15:**
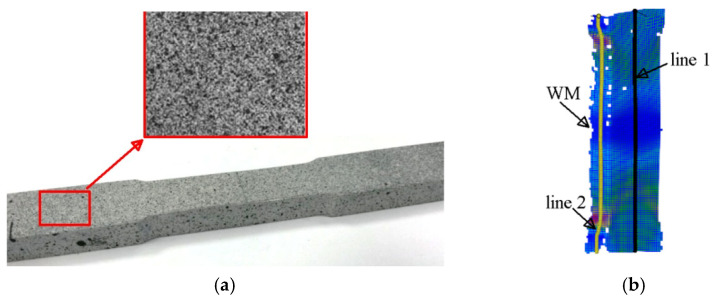
Specimen taken from the laser joint: (**a**) with the surface prepared for deformation measurement using the Aramis DIC system; (**b**) with measurement lines positioned on the side of the root—measurement line 1 (black) and in cross-section by thickness—measurement line 2 (yellow).

The test results presented in the previous sections prove that welded joints contain materials with different properties. Especially in laser-made joints, the difference in the mechanical properties of the material in different zones is significant. So, during loading, stress and strain fields of different intensities will be generated in different areas of the welded joints. This section presents tests made to assess the levels of strain and stress occurring in the material of welded joints during loading. Estimation of the stress and strain fields in the loaded joint was carried out with the ARAMIS digital image correlation system and using modelling and numerical calculations. In the first stage, strain fields were assessed using the Aramis digital image correlation system. Specimens were taken from the laser joints for uniaxial tensile testing. Tests were made on flat specimens in which the weld was positioned in the centre of the specimen. The face and ridge were removed, flush with the surface of the BM. The specimens were prepared by spraying a white primer and fine, highly dispersive black dots (Figure 15a). The specimens were stretched transverse to the weld axis. Using a video system, the strain fields on the surfaces of the analysed welded joints were determined. The strain values are presented as maps on the surface of the specimens and graphs generated along measurement line 1 (black) and measurement line 2 (yellow), which are schematically shown in Figure 15b.

**Figure 16:**
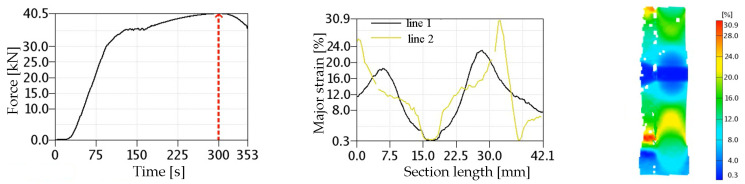
Examples of strain fields for a 4 L laser joint obtained with the Aramis video system at the time of reaching the ultimate tensile strength.

Example strain plots for the 4 L laser joint along measurement line 1 and measurement line 2, as well as the strain field at the specimen surface, are shown in Figure 16. All of the laser joints analysed had qualitatively similar strain distributions. In each loading step, the lowest levels of deformation during the uniaxial tensile test of the joints occurred in the WM and the HAZ. The specimens were loaded until the force dropped and constriction appeared, which occurred in the BM in all joints, demonstrating the high strength of the laser-welded joints.

**Figure 17:**
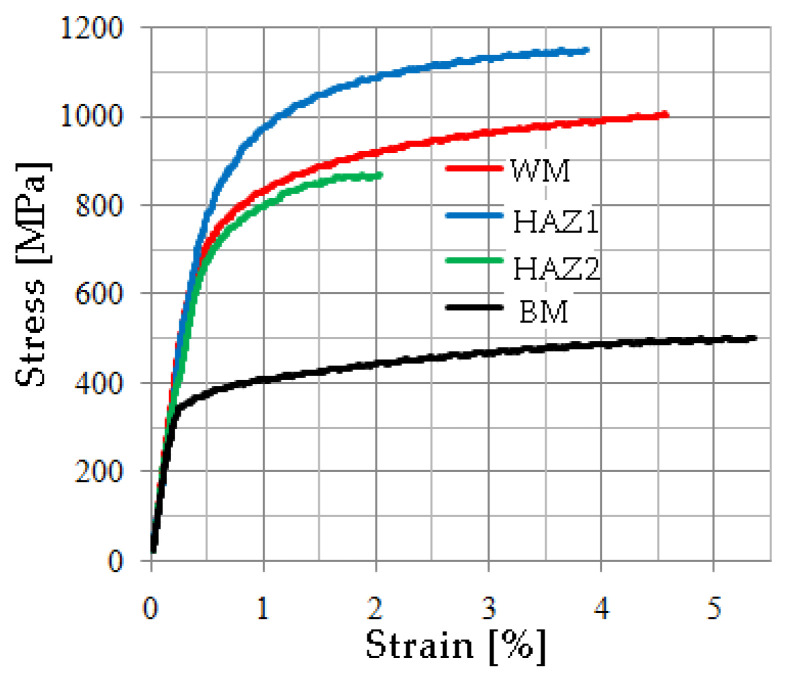
Stress–strain diagrams of the L2 joint zones.

In order to calculate the stress values occurring on surfaces where strain fields (or distributions) have been recorded, it is necessary to define the true stress–strain relationships of the material in the various areas of welded joints. For this purpose, data on the strength and ductility characteristics of materials from different welded joint zones, which were obtained during uniaxial tensile tests on the specimens (Section 3.3.1), were used. Examples of typical uniaxial tensile plots of materials from different zones for the 2 L laser joint are shown in Figure 17. The yield stress values for the stress–strain relationship are shown in Figure 17 and presented in Table 5. Numerical calculations were also carried out for the 2 L joint.

**Figure 18:**
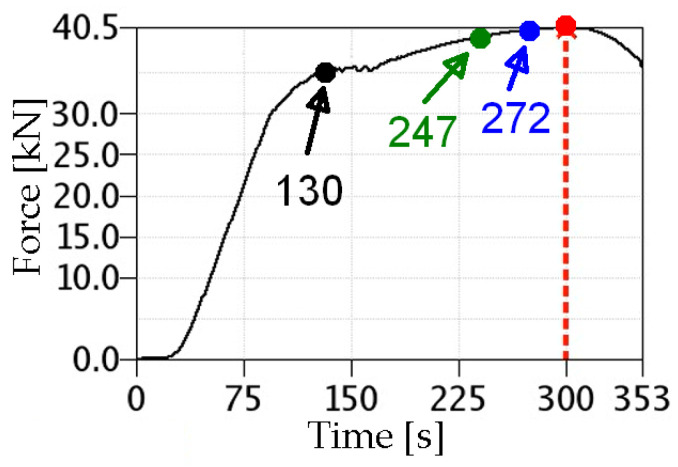
Plot of specimen loading over time (2 L joint, 13CrMo4-5 steel).

In order to determine the stress distribution inside the specimen, the laser-welded specimens were numerically modelled and then loaded with the same displacement as recorded during the experiment. The Abaqus CAE module version 6.12-3 was used for numerical calculations. Figure 18 presents a force–time diagram obtained for the 2 L specimen. Additionally, characteristic points are marked on the chart, for which the results obtained numerically and those recorded by the Aramis system will be compared later (numbers next to the points indicate time).

Four material zones were declared in the numerically modelled welded joint––WM, HAZ_1, HAZ_2, and BM––for which the corresponding true stress–strain relations were assumed (Figure 17). For numerical calculations, an elastic-plastic model with nonlinear hardening was used, and isotropic hardening was assumed.

**Figure 19:**
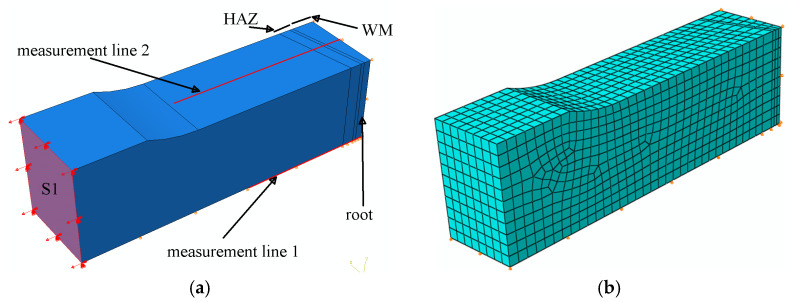
Numerical model of the laser joint: (**a**) with welding zones and measurement lines marked; (**b**) with FE mesh.

Due to the symmetry along the length and width of the specimen, numerical calculations were carried out on a model representing one-fourth of the laser joint. In the numerical model, loads were generated by applying an appropriate displacement value to the S1 surface (Figure 19a). The finite element mesh density was selected to match the measurement accuracy of the Aramis system. The mesh size ranged from 0.3 mm in HAZ_1 to 1 mm in HAZ_2 and the WM. Eight-node rectangular elements were used. The number of finite elements in the model was 3432. The finite element (FE) mesh is shown in Figure 19b.

**Figure 20:**
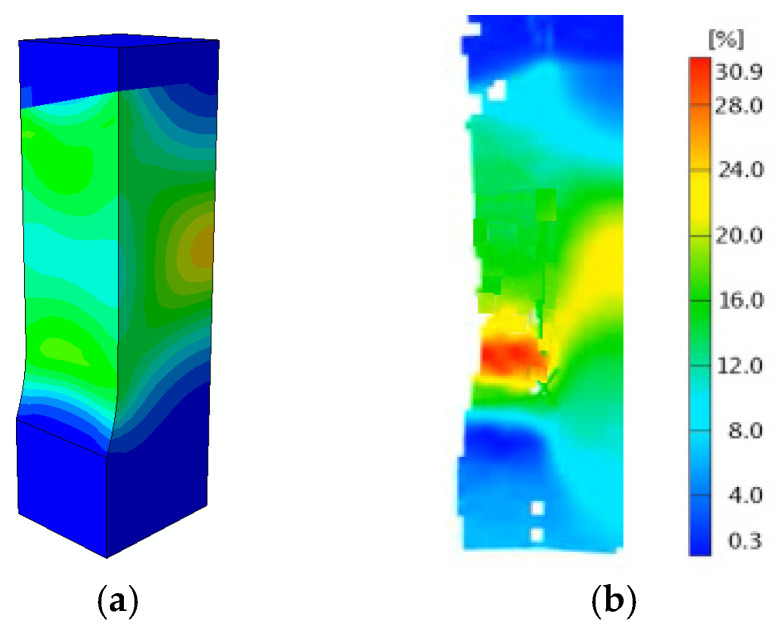
Strain field maps for the 2 L laser joint on the specimen surface: (**a**) obtained numerically; (**b**) obtained using the Aramis video system.

In order to verify the accuracy of the numerical model, the strain values calculated numerically were compared with the results obtained experimentally using the Aramis 3D 5M optical video system [27]. Figure 20a shows the strain field maps calculated using FEM, and Figure 20b shows the strain field maps obtained on the specimen using the Aramis video system when the joint reaches its maximum tensile strength (stage 300—see Figure 18). As can be seen, the levels and shapes of the strain fields recorded on the specimen surface are similar to each other. In both tests, areas of maximum strain were recorded in the BM of the specimen. The good agreement between the results of the strain fields obtained by the numerical and the experimental methods using a video system on the surface of the specimen allows us to conclude that the numerical model of the tested welded joint is made correctly.

**Figure 21:**
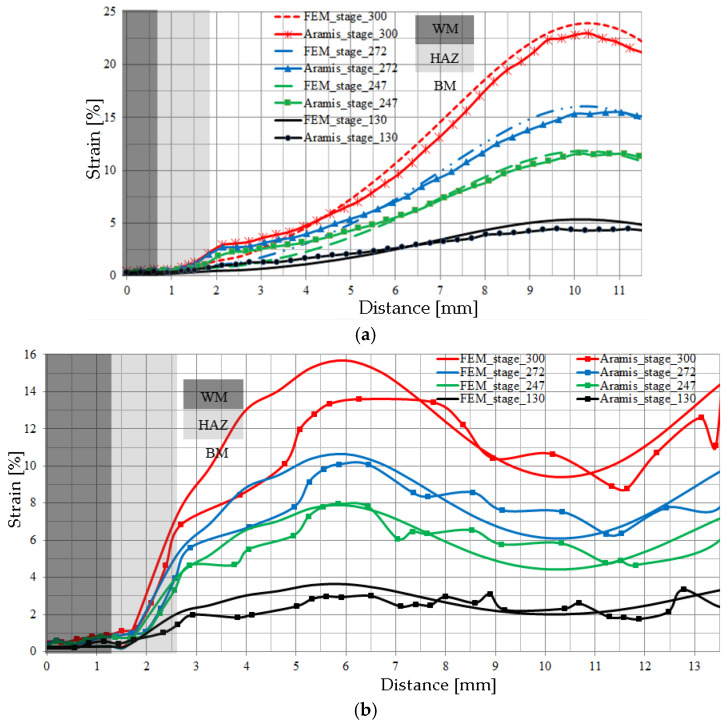
Strain distributions for given load steps in a laser-welded joint: (**a**) on the root side—measurement line 1; (**b**) in the middle of thickness—measurement line 2.

Figure 21a,b shows the results of deformation measurements obtained along line 1, located on the root side, and measurement line 2, located in the middle of the laser joint thickness, respectively. The lines without markers correspond to deformations calculated using FEM, while lines with markers correspond to deformations obtained using the Aramis video system. The distance 0 mm on the horizontal axis corresponds to the central part of the weld.

The area of lowest strain in the laser joint is in the WM. The strain values gradually increase in the HAZ material, reaching maximum values in the BM. For measurement line 1, the maximum strain level occurs at a distance of approximately 10 mm from the weld axis, while for measurement line 2 it occurs at a distance of approximately 6 mm. Measuring line 1, located on the root side of the WM, shows lower strain levels at the transition of the HAZ and BM than is the case for measuring line 2. Comparing the strain values obtained using FEM and those determined experimentally, one can observe good agreement between the strain values obtained. In the areas of the joint where the highest strain levels were recorded, it can be seen that the results obtained numerically are slightly higher than those obtained using the Aramis optical video system. The uniaxial tensile test of the joint was carried out until a significant narrowing appeared, which formed in the BM. Similarly, in the 1 L joint, narrowing was also observed in the BM.

**Figure 22:**
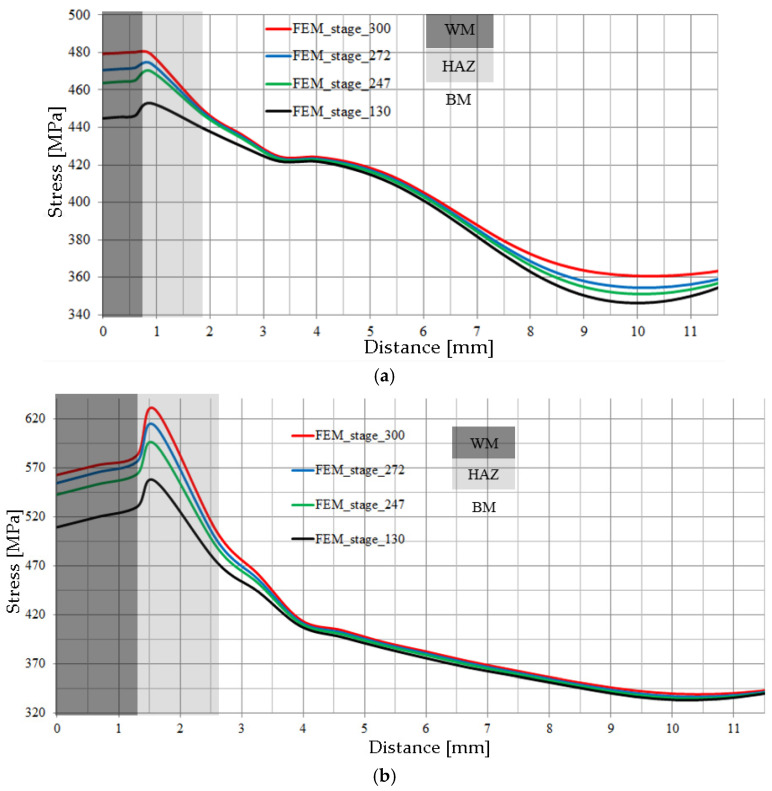
Stress distributions for the given load stages in laser-welded joint 2: (**a**) on the root side—measurement line 1; (**b**) in the middle of thickness—measurement line 2.

Figure 22a,b show the distributions of tensile stresses along measurement lines 1 and 2. High stress levels in the welded joint are found in the WM, then the stresses reach their maximum value in the HAZ and then gradually decrease to reach their minimum value in the BM. It should be noted that, for measurement line 2 located in the centre of the joint thickness, the stress level in the WM and the HAZ is higher (Figure 22b) than the stress level occurring along measurement line 1 located on the root side of the weld (Figure 22a). With increasing joint loading, there is an increase in the stress level in the WM and the HAZ for each of the measuring lines analysed.

The absolute stress values in the WM and HAZ zones are almost twice as high as in the BM. However, with regard to the level of yield stress values of the material from the respective material zone, the situation is different. The yield stress for the material in the WM and HAZ is high (*Re* > 700 MPa), while for BM the *Re* ≈ 350 MPa (Table 5). So, when the joint is loaded in the WM and HAZ, the stress level does not exceed the yield strength, then in BM it exceeds it, especially at a distance of 3–6 mm from the centre of the weld, resulting in the occurrence of narrowing in the specimen.

## 4. Discussion and Conclusions

Making a TIG-welded joint requires a number of preliminary steps, including proper preparation of the weld groove and preheating of the plates to be joined. The welding process itself, specifically the way the weld is formed, takes several beads and is therefore time-consuming and requires the use of filler material.

From the determined hardness distributions, it can be observed that all zones in the analysed welded joints have a higher hardness than the BM, which is also reflected in the determined strength properties—in the weld and in the HAZ they are higher than in the BM.

No cracks were observed in the bending tests carried out on the face and root sides of the welded joints, which confirms that the joints have the required ductility.

The fracture toughness tests carried out for TIG-made joints indicate that the WM and the material in the vicinity of the FL have a slightly lower critical value of fracture toughness compared to the BM. However, it is important to note that the material from all zones of the welded joint showed a ductile subcritical fracture growth mechanism during fracture toughness tests and a high critical value of fracture toughness (*K*_JC_ ≥ 200 MPa⋅m^1/2^). Values of *K*_JC_ > 100 MPa⋅m^1/2^ exclude the occurrence of a brittle fracture mechanism, according to FITNET [28].

Laser-welded joints are characterised by a higher hardness in the WM and the HAZ material than in TIG welding, which increases as the LWE level decreases. On the basis of the metallographic research carried out, it can be observed that laser-welded joints are characterised by a narrow weld and HAZ, in which different types of microstructure are observed, mainly of martensitic and bainitic types, which translates into high values in the hardness distributions and in the levels of strength characteristics in the different zones of the joint.

In bending tests of welded joints carried out on the face and root sides of the weld, no cracks were observed, which indicates a high level of ductility.

When testing the fracture toughness of the material from the different laser joint zones, positive results were generally obtained, with fracture toughness values higher than the reference level (*K*_JC_ ≥ 140 > 100 MPa⋅m^1/2^). The lowest fracture toughness values (*K*_JC_ = 140–155 MPa⋅m^1/2^) are found in WM made with a higher LWE level = 540 kJ/m. Lowering the LWE level leads to an increase in the critical fracture toughness values in all joint zones. Similar results for the dependence of fracture toughness on the LWE level were also obtained for joints made from high-strength S960Q steel [17,29].

Numerical modelling and load simulation of the welded joint made it possible to determine the areas where the highest levels of relative tensile stresses occur and where the material will deform plastically. For this purpose, the following was performed:

- A model of a welded joint that includes the WM, two areas in the HAZ and the BM. Material stress–strain relationships were developed for each material in the respective areas. These relationships were developed using correlation relationships between hardness and the relevant material strength characteristics.
- Joint loading was carried out by uniaxial tension and by numerical simulation. During the experiment, deformations on the specimen surface were recorded using an Aramis video system. Good agreement was observed between the strain fields obtained using the DIC (Digital Image Correlation) method and those calculated numerically, demonstrating the correctness of the numerical model used.
- The highest absolute values of numerically calculated tensile stresses were obtained in the HAZ zone immediately adjacent to the WM. Significantly, in the centre of the specimen the stress level is much higher than at the surface. However, in the WM and HAZ zones, the material has high yield stress values that exceed the tensile stresses, so plasticisation of the material does not occur here. In contrast, in the BM at the HAZ, the level of tensile stress exceeds the yield stress value, and it is here that plasticisation and formation of a neck was observed.

In conclusion, we can say that the numerical model presented in this paper allows strength assessment of welded joints to be carried out with good accuracy. What is necessary here is knowledge of the correlation relationship between hardness and material strength characteristics.

The presented method has also been successfully applied to the strength analysis of welded joints made of high-strength S960QC steel [17,30,31]. Compared to the method for evaluating the strength of welded joints presented in FITNET [32], which only considers the WM and BM strength characteristics, the developed approach takes into account the strength characteristics of the HAZ material in several areas, resulting in higher accuracy and measurability.

## Figures and Tables

**Table 1:** Mechanical properties and hardness of 13CrMo4-5 and 16Mo3 steels.

Steel	*R*_e_ [MPa]	*R*_m_ [MPa]	*A*_5min_ [%]	Hardness HV10
13CrMo4-5	290	440–590	22	145–165
16Mo3	280	450–600	22	140–160

**Table 2:** Chemical composition of 13CrMo4-5 and 16Mo3 steels (wt.%).

Steel	C	Si	Mn	P	S	Cr	Mo	Ni	N/V	Cu
13CrMo4-5	0.10–0.18	0.35	0.40–0.70	0.025	0.02	0.70–1.15	0.40–0.60	0.30	0.22–0.35	0.30
16Mo3	0.12–0.20	0.35	0.40–0.90	0.025	0.01	0.30	0.25–0.35	0.30	0.012	0.30

**Table 3:** Technological parameters of TIG welding.

Base Material	Joint	Bead No.	Rod Diameter [mm]	Interpass Temperature [°C]	Shielding Gas Flow [L/min]	Speed [mm/min]	LWE [kJ/m]	LWE_śr_ [kJ/m]
13CrMo4-5	1T	1	ϕ = 2.4	-	12	34	1790	1563
		2	ϕ = 2.4	238		58	1760	
		3	ϕ = 2.4	213		79	1530	
		4	ϕ = 2.4	273		102	1170	
16Mo3	2T	1	ϕ = 2.0	24	42	12	1630	1645
		2	ϕ = 2.4	72	59	12	1850	
		3	ϕ = 2.4	54	63	12	1740	
		4	ϕ = 2.4	110	88	12	1360	

**Table 4:** Technological parameters of laser welding.

Base Material	Joint	Beam Power [kW]	Speed [mm/min]	LWE [kJ/m]	Shielding Gas
13CrMo4-5	1 L	6	600	540	He + 3%O_2_
	2 L	6	700	463	He
16Mo3	3 L	6	600	540	He + 3%O_2_
	4 L	6	800	405	

**Table 5:** Yield strength at individual L2 joint zones.

Joint Zone	WM	HAZ_1	HAZ_2	BM
*R*_e_ [MPa]	780	890	720	370

## Data Availability

The original contributions presented in the study are included in the article, further inquiries can be directed to the corresponding author.

## Nomenclature

Symbols		
*a*	[mm]	crack length
*A*_5_	[%]	elongation
*B*	[mm]	specimen thickness
*E*	[GPa]	Young’s modulus
*J*	[kN/m]	J integral
*J*_IC_	[kN/m]	critical value of the J integral
*K*_JC_	[MPa·m^1/2^]	critical value of the stress intensity factor
LWE	[kJ/m]	linear welding energy
LWE_śr_	[kJ/m]	average linear welding energy
*R*_e_	[MPa]	yield strength
*R*_m_	[MPa]	ultimate tensile strength
*ν*	[-]	Poisson’s ratio
*W*	[mm]	specimen height
Abbreviations		
ASTM	American Society for Testing and Materials	
AHSS	Advanced high-strength steel	
BM	Base material	
FL	Fusion line	
FEM	Finite element method	
HAZ	Heat-affected zone	
HAZ_1	Heat-affected zone at fusion line	
HAZ_2	Heat-affected zone of the base material	
MMA	Manual metal arc welding	
SAW	Submerged arc welding	
SENB	Single edge notch in bending	
TIG	Tungsten inert gas	
WM	Weld material	
